# Predictors of Biliary Drainage Dysfunction Following EUS‐Guided Gallbladder Drainage for Malignant Distal Biliary Obstruction: A Multicenter Retrospective Study: GALLBLADEUS‐2

**DOI:** 10.1111/den.70211

**Published:** 2026-07-14

**Authors:** M. Goudot, D. Lorenzo, L. Caillo, J. Privat, S. Ouazana, S. Leblanc, B. Napoléon, J. Jacques, F. Moryoussef, J. F. Bourgaux, R. Gérard, C. Loras Alastruey, S. Bazaga, J. Daniel, J. Albouys, N. Williet, M. Schaefer, A. Benezech, M. Camus‐Duboc, T. Wallenhorst, P. Mayer, J. Gornals, T. Mura, A. Debourdeau

**Affiliations:** ^1^ Department of Gastroenterology University Hospital of Nimes, University of Montpellier Nimes France; ^2^ Sorbonne Université Centre de Recherche Saint Antoine (CRSA) & Hôpital Saint Antoine, APHP Paris France; ^3^ CHU Nîmes Nîmes France; ^4^ Vichy Hospital Vichy France; ^5^ Hôpital Privé Jean Mermoz Lyon France; ^6^ Limoges University Hospital Limoges France; ^7^ Poissy‐Saint‐Germain Hospital Poissy France; ^8^ CHU Lille Service des Maladies de l'Appareil Digestif Lille France; ^9^ Hospital Universitari Mútua de Terrassa, Terrassa Barcelona Spain; ^10^ Hospital General de Granollers Granollers Spain; ^11^ Clinique Saint Jean Sud de France Saint Jean de Vedas France; ^12^ Saint‐Etienne University Hospital Saint‐Etienne France; ^13^ Nancy University Hospital Nancy France; ^14^ Avignon Hospital Avignon France; ^15^ Rennes University Hospital Rennes France; ^16^ Strasbourg University Hospital Strasbourg France; ^17^ Bellvitge University Hospital, University of Barcelona, Bellvitge Biomedical Research Institute (IDIBELL) Barcelona Spain; ^18^ Department of Biostatistics Epidemiology and Public Health, University Hospital Centre Nimes Nimes France; ^19^ INSERM U1298—Montpellier Neuroscience Institute Montpellier France; ^20^ CHU de Montpellier Montpellier France

**Keywords:** drainage, endoscopic ultrasound, gallbladder, lumen‐apposing metal stent, pancreatic cancer

## Abstract

**Objectives:**

Endoscopic ultrasound‐guided gallbladder drainage is increasingly used for malignant distal biliary obstruction, yet predictors of drainage dysfunction remain unknown. This study aimed to identify clinical and procedural factors associated with dysfunction, clinical success, and morbidity.

**Methods:**

This retrospective, international, multicenter cohort study included consecutive patients who underwent endoscopic ultrasound‐guided gallbladder drainage with electrocautery‐enhanced lumen‐apposing metal stents for malignant distal biliary obstruction across 17 centers (March 2017–September 2024). The primary endpoint was biliary drainage dysfunction analyzed using Kaplan–Meier estimation and Cox regression. Secondary endpoints included predictors of clinical success and high‐grade adverse events.

**Results:**

A total of 166 patients were included. Technical and clinical success rates were 98.8% (164/166) and 82.9% (136/164), respectively. Among 146 patients in the survival analysis, 12‐month biliary patency was 70.4%. Neither the access route (transgastric 68.5% vs. transduodenal 73.5%, *p* = 0.50) nor stent diameter (8, 10, or 15 mm; *p* = 0.82) influenced patency. Ascites was the only independent predictor of dysfunction (HR 2.45 [95% CI 1.17–5.13], *p* = 0.018). Endoscopic ultrasound‐confirmed cystic duct patency was independently associated with clinical success (86.7% vs. 69.4%, OR 2.88 [95% CI 1.19–6.96], *p* = 0.019). The 15‐mm stent was associated with fewer high‐grade adverse events (14.3% vs. 32.9%, OR 0.31 [95% CI 0.14–0.69], *p* = 0.004) without affecting patency.

**Conclusions:**

In this large multicenter series, neither access route nor stent diameter influenced biliary patency. Ascites was the sole independent predictor of dysfunction. A protective signal of 15 mm stents against high‐grade morbidity warrants prospective confirmation.

## Graphical abstract

This multicenter study identified predictors of drainage dysfunction after EUS‐guided gallbladder drainage for malignant distal biliary obstruction. Ascites emerged as the main independent predictor of unfavorable outcome, whereas the route of access and LAMS diameter had no significant impact on the primary composite endpoint. These findings support EUS‐GBD as a viable rescue strategy while highlighting patient‐level factors that outweigh technical choices.

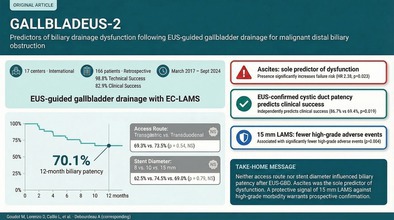

## 1 Introduction

Malignant distal biliary obstruction (MDBO) complicates 70%–80% of pancreatic head cancers, frequently delaying chemotherapy and impairing quality of life [1]. Endoscopic retrograde cholangiopancreatography (ERCP) remains the first‐line approach for biliary decompression [2], but fails in 10%–20% of cases due to duodenal invasion, papillary encasement, or prior duodenal stenting [3].

In the setting of ERCP failure, endoscopic ultrasound‐guided biliary drainage (EUS‐BD) has emerged as a preferred alternative to percutaneous transhepatic biliary drainage (PTBD) [3, 4]. EUS‐guided choledochoduodenostomy (EUS‐CDS) and hepaticogastrostomy (EUS‐HGS) are the most established techniques, but both require specific anatomical prerequisites [5]. EUS‐CDS requires adequate common bile duct diameter (typically > 15 mm) and stable endoscopic positioning, while EUS‐HGS requires sufficient intrahepatic duct dilation and carries higher risk in patients with ascites. When neither is feasible—due to small‐caliber CBD, extensive duodenal invasion, insufficient intrahepatic dilation, or significant ascites—EUS‐guided gallbladder drainage (EUS‐GBD) using electrocautery‐enhanced lumen‐apposing metal stents (EC‐LAMS) has gained traction as a rescue strategy [6, 7], and has been shown to be effective as a first‐line strategy as well [8, 9].

EUS‐GBD for MDBO demonstrates excellent technical (99%–100%) and clinical (85%–89%) success rates, with adverse event rates of 10%–14% [8, 9, 10]. However, predictors of long‐term dysfunction remain unexplored. The ESGE guideline favors the transduodenal approach for acute cholecystitis [3], but this is less applicable to MDBO, and the i‐EUS Delphi consensus [11] recognized that route selection should be individualized, highlighting a lack of data in jaundiced patients. In contrast to EUS‐CDS, where dysfunction has been linked to stent‐duct misalignment [12, 13], the determinants of long‐term patency after EUS‐GBD remain unknown.

The aim of this study was to identify clinical and procedural predictors of drainage strategy failure following EUS‐GBD for MDBO in a large international multicenter cohort, encompassing both early clinical failure and delayed biliary obstruction.

## 2 Methods

### 2.1 Study Design and Ethics

This retrospective, international, multicenter cohort study included consecutive patients who underwent EUS‐GBD for MDBO from 17 centers (15 in France, 3 in Spain) between March 2017 and September 2024. Data were extracted from a prospectively maintained registry. The study followed the STROBE guidelines and received institutional review board approval (IRB‐MTP_2022_07_202201185). Given the retrospective design, informed consent was waived.

### 2.2 Study Population

Inclusion criteria were age ≥ 18 years, confirmed MDBO, EUS‐GBD performed using EC‐LAMS (Hot AXIOS, Boston Scientific), and minimum follow‐up of 30 days or earlier death.

Exclusion criteria were benign strictures, proximal biliary obstruction, follow‐up < 30 days in surviving patients, and EUS‐GBD for acute cholecystitis without biliary drainage intent.

Approximately half the patients overlap with the CHOLEBLADEUS study [14] (EUS‐GBD vs. EUS‐CDS), which addresses a different research question.

### 2.3 Intervention and Data Collection

EUS‐GBD was performed using a linear echoendoscope under deep sedation or general anesthesia. The gallbladder was punctured under real‐time EUS guidance via a transgastric or transduodenal approach, based on anatomical suitability. EC‐LAMS were deployed using the electrocautery‐enhanced free‐hand technique in 162 patients (97.6%) and guidewire‐assisted technique in 4 (2.4%); available diameters were 8, 10, and 15 mm. A coaxial double‐pigtail stent was placed at the endoscopist's discretion. Cystic duct patency was assessed by EUS when technically feasible, though not systematically across all centers. In a subset of patients, EUS‐GBD was performed as a first‐line approach without prior ERCP, based on prospective data supporting its efficacy in palliative MDBO [8], particularly when the common bile duct was below 14–15 mm or duodenal invasion was anticipated. Antibiotic prophylaxis followed local protocols. Gallbladder stones were not an absolute contraindication. Data were collected retrospectively via a standardized electronic case report form.

### 2.4 Outcome Definitions

#### 2.4.1 Primary Endpoint

The primary endpoint was the occurrence of biliary drainage dysfunction following EUS‐GBD, defined as a composite time‐to‐event endpoint designed to capture both early dysfunction (reflecting inadequate biliary decompression within the first 30 days) and delayed dysfunction (reflecting secondary biliary obstruction after initial clinical success). Each patient was assigned a dysfunction date as follows:

– *In patients achieving clinical success:* the dysfunction date was defined as the date of recurrent biliary obstruction (rise in serum bilirubin above twice the post‐nadir value and/or clinical signs of cholangitis) requiring endoscopic, radiological, or surgical reintervention.
– *In patients not achieving clinical success:* the dysfunction date was defined as the date of endoscopic reintervention within 30 days, or day 30 post‐procedure if no reintervention was attempted.
– *In patients without dysfunction:* patients were censored at the date of death or last follow‐up contact, whichever occurred last.

#### 2.4.2 Secondary Endpoints

Clinical success was defined as a > 50% decrease in total bilirubin levels at day 7 or normalization at day 30 (< 48 μmol/L). This definition, previously used in the CHOLEBLADEUS study, was applied consistently throughout all data extraction and statistical analyses; no reanalysis was required. When bilirubin values were not available at the exact prespecified timepoints, clinical success was adjudicated using the closest available measurement within a clinically acceptable window.

Technical success was defined as successful EC‐LAMS deployment with confirmed positioning, with bile drainage through the stent.

Adverse events were graded per the ASGE lexicon [15] and the AGREE classification [16]. High‐grade morbidity was defined as AGREE ≥ IIIa. For the descriptive analysis of adverse events, delayed events were further stratified as early delayed (24 h to 30 days) and late (> 30 days) to distinguish procedure‐related complications from stent‐related events. High‐grade morbidity was defined as any adverse event graded AGREE ≥ IIIa, irrespective of timing, in order to capture both procedure‐related and stent‐related complications over the entire follow‐up period.

### 2.5 Statistical Analysis

Categorical variables were expressed as frequencies and percentages, and continuous variables as mean ± SD or median [IQR] according to distribution (Shapiro–Wilk test). Biliary drainage dysfunction was analyzed using Kaplan–Meier estimation with log‐rank comparisons. Pairwise comparisons between LAMS diameter subgroups were performed using separate log‐rank tests. Univariate Cox regression was performed for all potential predictors. Three multivariable Cox models were pre‐specified (Table 3): Model A (primary) included ascites, access route, and LAMS diameter; Model B the three best univariate predictors; Model C was exploratory and included clinical success, acknowledging its role in the composite endpoint. Models were restricted to three covariates (~10 events per variable [16]). A sensitivity analysis restricted to clinical success patients evaluated predictors of late dysfunction independently from early failure. Predictors of clinical success and high‐grade morbidity (AGREE ≥ IIIa) were assessed by logistic regression. Nineteen patients with incomplete follow‐up and one with a data inconsistency (composite endpoint date preceding the inclusion date) were excluded from time‐to‐event analyses but included in cross‐sectional analyses. All tests were two‐sided (*p* < 0.05). Statistical analyses were performed using Python 3.12 and EasyMedStat (www.easymedstat.com).

## 3 Results

### 3.1 Study Population and Baseline Characteristics

**FIGURE 1:**
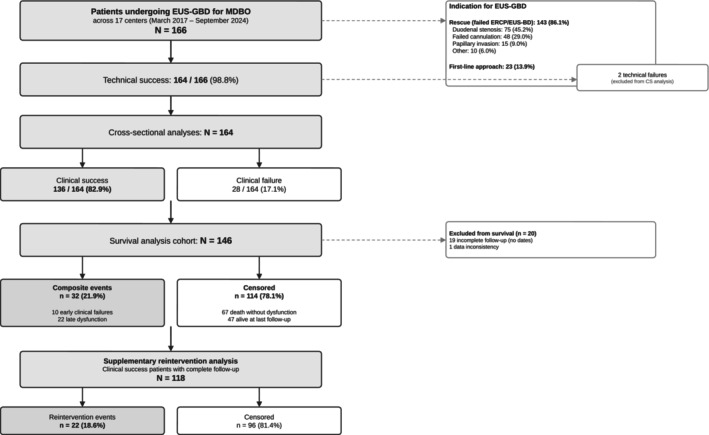
Study flow diagram. CS, clinical success; ERCP, endoscopic retrograde cholangiopancreatography; EUS‐GBD, endoscopic ultrasound‐guided gallbladder drainage; FU, follow‐up; MDBO, malignant distal biliary obstruction.

Between March 2017 and September 2024, 166 patients underwent EUS‐GBD for MDBO across 17 centers. Technical success was achieved in 164 (98.8%). After excluding 19 patients with incomplete follow‐up and 1 data inconsistency, 146 were available for the primary survival analysis (Figure 1). The complete cohort (*N* = 166) was used for baseline description. Cross‐sectional analyses of clinical success and adverse events were performed on the 164 technically successful patients (Figure 1).

Mean age was 71.8 ± 12.2 years (49.4% male). Pancreatic adenocarcinoma was the underlying malignancy in 71.7% (119/166), followed by other malignancies (15.1%), metastatic lymphadenopathy (8.4%), and cholangiocarcinoma (4.8%). Most patients had metastatic (54.8%) or locally advanced (23.5%) disease. ECOG ≥ 3 was present in 38.0%, duodenal stenosis in 51.2%, and ascites in 36.1%. The therapeutic plan was chemotherapy in 54.8%, palliative care in 37.3%, and surgery in 6.0% (*n* = 10; 7/10 underwent surgery). EUS‐GBD was performed as rescue after failed ERCP/EUS‐BD in 86.1% and first‐line in 13.9%. Among the 143 rescue cases, the specific reasons were duodenal stenosis (*n* = 75, 52.4%), failed cannulation (*n* = 48, 33.6%), papillary invasion (*n* = 15, 10.5%), and other (*n* = 5, 3.5%). The transgastric route was used in 66.9% (111/166). LAMS diameters were 15 mm (50.6%), 10 mm (34.3%), and 8 mm (13.9%). Two patients with technical failure had undetermined LAMS diameter. A coaxial double‐pigtail stent was placed in 17.5%. Cystic duct patency was verified by EUS in 77.7% (Table 1).

**TABLE 1:** Baseline characteristics of the study population (*N* = 166).

Variables	Category	*N* = 166
*Demographics*		
Age, years, mean ± SD		71.8 ± 12.6
Male sex		82 (49.4%)
ECOG performance status		
0–1		40 (24.1%)
2		60 (36.1%)
3–4		63 (38.0%)
NC		3 (1.8%)
*Disease characteristics*		
Cause of MBO		
Pancreatic adenocarcinoma		119 (71.7%)
Cholangiocarcinoma		8 (4.8%)
Metastatic lymphadenopathy		14 (8.4%)
Other		25 (15.1%)
Disease stage		
Resectable		13 (7.8%)
Borderline resectable		17 (10.2%)
Locally advanced		39 (23.5%)
Metastatic		91 (54.8%)
NC		6 (3.6%)
Duodenal stenosis		85 (51.2%)
Ascites		60 (36.1%)
Therapeutic plan		
Chemotherapy		91 (54.8%)
Palliative care		62 (37.3%)
Surgery		10 (6.0%)
NC		3 (1.8%)
*Procedural characteristics*		
Indication		
Rescue (failed ERCP/EUS‐BD)		143 (86.1%)
First‐line approach		23 (13.9%)
Access route		
Transgastric		111 (66.9%)
Transduodenal		55 (33.1%)
LAMS diameter		
8 mm		23 (13.9%)
10 mm		57 (34.3%)
15 mm		84 (50.6%)
Free‐hand technique		162 (97.6%)
Coaxial double‐pigtail stent		29 (17.5%)
Cystic duct patency verification		
EUS‐checked		129 (77.7%)
Imaging‐checked (CT/MRI)		12 (7.2%)
No mention of cystic duct assessment		25 (15.1%)
Gallbladder stones		32 (19.3%)
CBD diameter, mm, mean ± SD		13.2 ± 3.6 (*n* = 133)
*Outcomes*		
Technical success		164 (98.8%)
Clinical success		137/164 (83.5%)
Bilirubin, μmol/L, median [IQR]		
Baseline		147 [73–247] (*n* = 141)
Day 7		57 [24–113] (*n* = 123)
Day 30		22 [11–45] (*n* = 100)

*Note:* Continuous variables: mean ± SD or median [IQR]. Categorical: *n* (%).

Abbreviation: NC = not collected.

### 3.2 Clinical and Technical Success and Primary Endpoint

Clinical success was achieved in 136/164 patients (82.9%). Median bilirubin decreased from 148 μmol/L [IQR 74.5–243.5] at baseline to 58.3 μmol/L [24.8–115.0] at day 7 and 22.0 μmol/L [11.0–45.0] at day 30.

**FIGURE 2:**
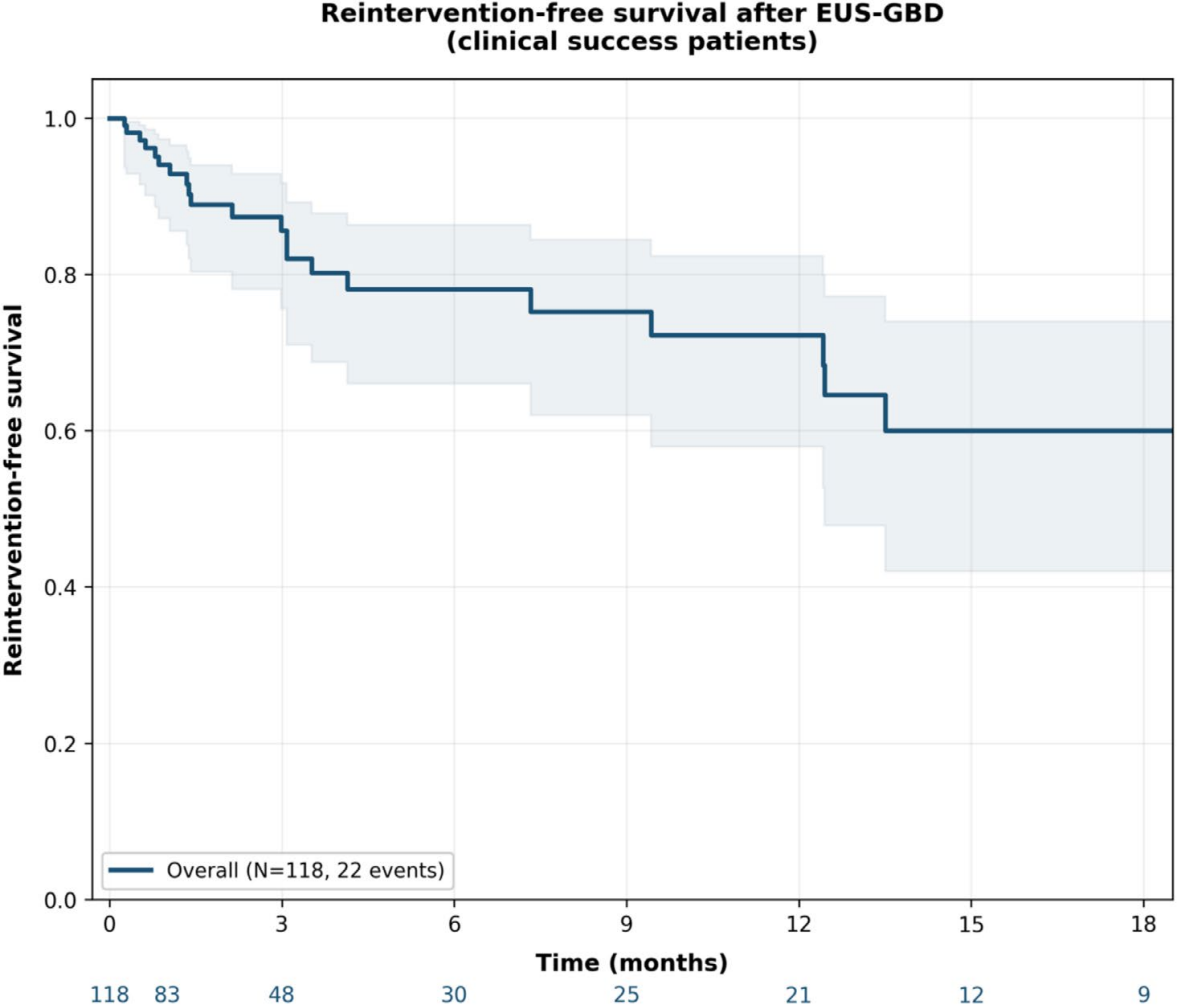
Kaplan–Meier curve of biliary patency following EUS‐GBD (*N* = 146). Numbers at risk are shown below the curve.

Median follow‐up was 1.6 months [IQR 0.5–7.8]; 88 patients (53.0%) had died at last contact. Among 146 patients in the survival analysis, 32 composite events occurred (21.9%): 10 early failures and 22 late dysfunction events (median follow‐up among censored patients: 72 days). The overall median biliary patency was not reached; Biliary patency rates were 87.7% at 1 month, 80.9% at 3 months, 74.9% at 6 months, and 70.4% at 12 months (Figure 2).

#### 3.2.1 Impact of Access Route and LAMS Diameter

**FIGURE 3:**
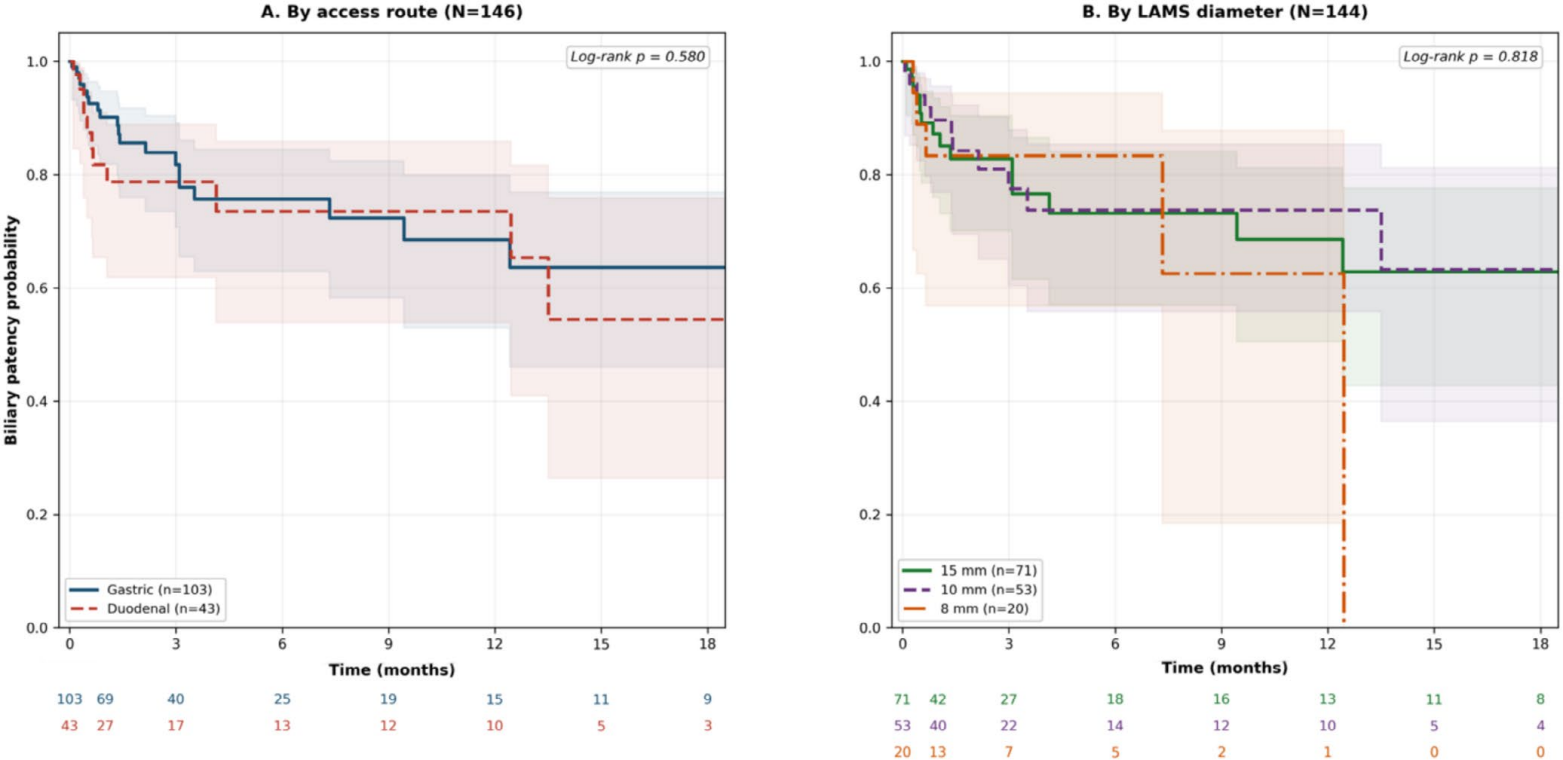
Kaplan–Meier curves of composite biliary dysfunction free‐survival stratified by (A) access route (transgastric vs. transduodenal) and (B) LAMS diameter (8 mm vs. 10 mm vs. 15 mm).

No difference in biliary patency was observed between transgastric and transduodenal approaches (12‐month: 68.5% [95% CI 55.0–82.6] vs. 73.5% [57.7–89.3], log‐rank *p* = 0.50; HR 0.80 [95% CI 0.38–1.66]; Figure 3A), confirmed in the clinical success subgroup (HR 1.22, *p* = 0.67). When analyzed separately, neither early clinical failure (transgastric 16.4% vs. transduodenal 16.7%, *p* = 1.00) nor late dysfunction among responders (16.3% vs. 15.6%, *p* = 1.00) differed between routes (Table S1).

LAMS diameter did not influence biliary patency (12 months: 62.5%, 73.8%, and 68.6% for 8, 10, and 15 mm; 3‐group log‐rank *p* = 0.82; Figure 3B). No pairwise comparison reached significance. No other procedural variable was associated with composite dysfunction: coaxial double‐pigtail (HR 1.16, *p* = 0.74), first‐line versus rescue (HR 0.76, *p* = 0.61), EUS‐confirmed cystic duct patency (HR 1.29, *p* = 0.60), duodenal stenosis (HR 1.01, *p* = 0.98), or gallstones (HR 1.47, *p* = 0.39) (Table 2).

**TABLE 2:** Univariate analysis of factors associated with biliary drainage dysfunction—Primary outcome (Cox proportional hazards, *N* = 146).

Variables	N	Ev.	Rate (%)	HR [95% CI]	*p* (LR)	*p* (Cox)
*Procedural factors*						
Gastric route (vs. duodenal)	103	21	20.4	0.81 [0.39–1.69]	0.54	0.54
LAMS 15 mm (vs. ≤ 10 mm)	72	15	20.8	0.92 [0.46–1.84]	0.81	0.81
First‐line (vs. rescue)	22	4	18.2	0.76 [0.27–2.16]	0.61	0.60
Cystic duct EUS‐checked	115	27	23.5	1.29 [0.50–3.37]	0.60	0.60
Double‐pigtail stent	28	6	21.4	1.16 [0.48–2.84]	0.74	0.74
Gallstones	24	6	25.0	1.47 [0.60–3.58]	0.39	0.40
*Patient/disease factors*						
**Ascites**	**47**	**13**	**27.7**	**2.62 [1.27–5.41]**	**0.007**	**0.009**
**Clinical success**	**118**	**22**	**18.6**	**0.42 [0.20–0.90]**	**0.023**	**0.025**
Pancreatic ADK (vs. other)	107	20	18.7	0.56 [0.27–1.15]	0.11	0.12
Duodenal stenosis	74	16	21.6	1.01 [0.50–2.02]	0.98	0.98
Metastatic stage	77	18	23.4	1.41 [0.70–2.84]	0.34	0.34
Male sex	74	17	23.0	0.98 [0.49–1.96]	0.95	0.95
ECOG ≥ 3	49	10	20.4	0.89 [0.40–1.96]	0.77	0.77
*Continuous variables (per unit)*						
Age (per year)	146			1.00 [0.97–1.03]		0.90
Bilirubin pre (per μmol/L)	135			1.00 [1.00–1.00]		0.51
CBD diameter (per mm)	120			0.99 [0.90–1.10]		0.93

*Note:* Bold: *p* < 0.05.

Abbreviations: Ev., events; LR, log‐rank; *N*, patients with factor present.

### 3.3 Predictors of Dysfunction

On univariate Cox regression, variables with *p* < 0.20 were as follows: ascites (HR 2.62, 95% CI 1.27–5.41, *p* = 0.007), clinical success (HR 0.42, 0.20–0.90, *p* = 0.023), and pancreatic adenocarcinoma (HR 0.56, 0.27–1.15, *p* = 0.11). In the primary multivariable model (Model A: ascites, access route, LAMS diameter), ascites was the only independent predictor of dysfunction (HR 2.45, 95% CI 1.17–5.13, *p* = 0.018), while access route (HR 0.79, *p* = 0.53) and LAMS diameter (HR 0.97, *p* = 0.93) were not significant. In Model B (best univariate predictors), ascites remained significant (HR 2.31, *p* = 0.028) while pancreatic adenocarcinoma showed a borderline association (HR 0.50, *p* = 0.063). In the exploratory Model C including clinical success, ascites remained the only significant predictor (HR 2.38, *p* = 0.023; Table 3). In the sensitivity analysis restricted to clinical success patients (CS+) (*n* = 118, 22 late events), no variable was significantly associated with late dysfunction. A supplementary reintervention analysis (*N* = 118 CS+ patients, 22 events) showed reintervention‐free survival of 85.6% at 3 months and 72.2% at 12 months, with no significant predictor (Tables S4 and S5 and Figures S2 and S3).

**TABLE 3:** Multivariable Cox regression models for biliary drainage dysfunction.

Variables	HR [95% CI]	*p*
*Model A:* *A priori clinical + procedural (N = 146, 32 events)*		
**Ascites**	2.45 [1.17–5.13]	0.018
Gastric route	0.79 [0.37–1.66]	0.53
LAMS 15 mm	0.97 [0.48–1.96]	0.93
*Model B: Best univariate predictors (N = 146, 32 events)*		
**Ascites**	2.31 [1.10–4.86]	0.028
Pancreatic ADK	0.50 [0.24–1.04]	0.063
Gastric route	0.79 [0.37–1.66]	0.53
*Model C: Exploratory, with clinical success* *(N = 146, 32 events)*		
**Ascites**	2.38 [1.12–5.03]	0.023
Clinical success	0.53 [0.24–1.16]	0.11
Gastric route	0.78 [0.37–1.62]	0.50

### 3.4 Predictors of Clinical Success

**FIGURE 4:**
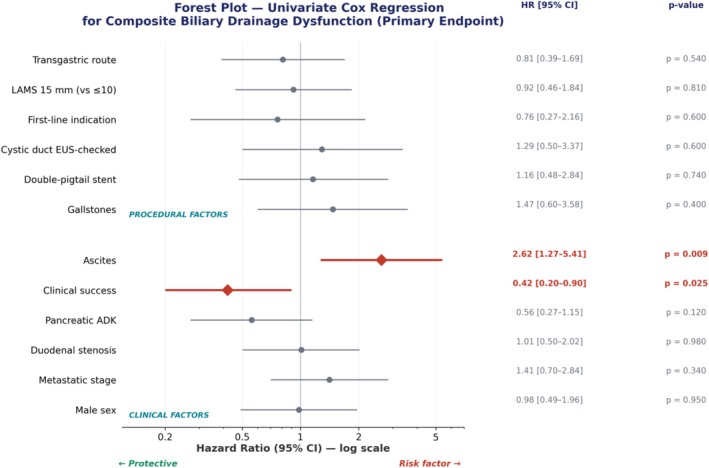
Forest plot of univariate Cox regression hazard ratios for biliary drainage dysfunction.

EUS‐confirmed cystic duct patency was the strongest procedural predictor of clinical success (86.7% vs. 69.4%, OR 2.87, 95% CI 1.20–6.88, *p* = 0.018), remaining significant after adjustment for access route and LAMS diameter (OR 2.88, 95% CI 1.19–6.96, *p* = 0.019; Table 4). Ascites was negatively associated with clinical success (75.0% vs. 87.5%, OR 0.43, *p* = 0.044). In the full multivariable model, cystic duct patency (OR 4.21, 95% CI 1.00–17.75, *p* = 0.050), duodenal stenosis (OR 2.83, 1.10–7.29, *p* = 0.031), and ascites (OR 0.33, 0.13–0.88, *p* = 0.026) were independently associated with clinical success (Figure 4).

**TABLE 4:** Predictors of clinical success (*N* = 164 patients with technical success).

Variables	CS+ *n*/N	CS– n/N	CS rate	OR [95% CI]	*p* (Fish.)	*p* (LR)
*Procedural factors*						
**Cystic duct EUS‐checked**	111/128	17/128	86.7%	**2.87 [1.20–6.88]**	**0.023**	**0.018**
Gastric route	92/110	18/110	83.6%	1.16 [0.50–2.72]	0.83	0.73
LAMS 15 mm	73/84	11/84	86.9%	1.79 [0.78–4.11]	0.21	0.17
Double‐pigtail	27/29	2/29	93.1%	3.22 [0.72–14.4]	0.17	0.13
First‐line	18/23	5/23	78.3%	0.70 [0.24–2.08]	0.55	0.52
Gallstones	27/32	5/32	84.4%	1.14 [0.40–3.27]	1.00	0.81
*Patient/disease factors*						
Ascites (present)	45/60	15/60	**75.0%**	**0.43 [0.19–0.98]**	0.052	**0.044**
Duodenal stenosis	74/84	10/84	88.1%	2.15 [0.92–4.99]	0.096	0.076
Male sex	65/80	15/80	81.2%	0.79 [0.35–1.79]	0.68	0.58
Pancreatic ADK	97/117	20/117	82.9%	0.99 [0.40–2.45]	1.00	0.99
ECOG ≥ 3	52/62	10/62	83.9%	1.11 [0.48–2.60]	0.83	0.80
*Multivariable model A (key procedural)*						
**Cystic duct EUS‐checked**				**2.88 [1.19–6.96]**		**0.019**
Gastric route				1.21 [0.50–2.91]		0.67
LAMS 15 mm				1.73 [0.74–4.04]		0.20
*Multivariable model B (all p < 0.20)*						
**Cystic duct EUS‐checked**				**4.21 [1.00–17.8]**		**0.050**
**Duodenal stenosis**				**2.83 [1.10–7.29]**		**0.031**
**Ascites (presence)**				**0.33 [0.13–0.88]**		**0.026**

*Note:* Bold: *p* < 0.05. N = 164 (technical success).

Abbreviations: CS, clinical success; Fish, Fisher exact test; LR, logistic regression.

### 3.5 Adverse Events and Predictors of Cumulative High‐Grade Morbidity (AGREE≥IIIa)

Periprocedural adverse events (< 24 h) occurred in 10 patients (6.0%). Early delayed events (24 h to 30 days) occurred in 20 patients (12.0%). Late adverse events (> 30 days), predominantly stent obstruction (8/11), occurred in 11 patients (6.6%).

Cholangitis during follow‐up was documented in 22 patients (13.3%). High‐grade morbidity (AGREE ≥ IIIa) was observed in 39 patients (23.5%), predominantly as delayed events (*n* = 36, 21.7%) (Table 5).

**TABLE 5:** Adverse events and AGREE classification (*N* = 166).

	Periprocedural (< 24 h)	Delayed (> 24 h)	Total
*Adverse events*			
Any adverse event	10 (6.0%)	33 (19.9%)	43 (25.9%)
Cholangitis	—	22 (13.3%)	22 (13.3%)
*Adverse event types (detail)*			
Perforation/migration	5 (3.0%)	—	5 (3.0%)
Bleeding	1 (0.6%)	2 (1.2%)	3 (1.8%)
Periprocedural sepsis	2 (1.2%)	—	2 (1.2%)
Stent obstruction	—	12 (7.2%)	12 (7.2%)
Nonobstructive cholangitis	—	10 (6.0%)	10 (6.0%)
Stent migration	—	4 (2.4%)	4 (2.4%)
Other	2 (1.2%)	5 (3.0%)	7 (4.2%)
*AGREE classification*			
No adverse event	157 (94.6%)	125 (75.3%)	
Grade I	1 (0.6%)	1 (0.6%)	2 (1.2%)
Grade II	2 (1.2%)	4 (2.4%)	6 (3.6%)
Grade IIIa	4 (2.4%)	29 (17.5%)	33 (19.9%)
Grade IIIb	2 (1.2%)	4 (2.4%)	6 (3.6%)
Grade IVa	—	1 (0.6%)	1 (0.6%)
Grade IVb	—	1 (0.6%)	1 (0.6%)
Grade V	—	1 (0.6%)	1 (0.6%)
*Summary*			
Any AGREE ≥ IIIa	6 (3.6%)	36 (21.7%)	39 (23.5%)

On multivariable analysis, 15 mm LAMS was independently protective against high‐grade events (14.3% vs. 32.9%; OR 0.31, 95% CI 0.14–0.69, *p* = 0.004) and the transgastric route was borderline significant (OR 2.44, 0.99–5.97, *p* = 0.051; Table 6). Notably, the 15 mm LAMS was associated with fewer high‐grade events without any detrimental impact on biliary patency, suggesting a dissociation between the morbidity profile and long‐term drainage function.

**TABLE 6:** Predictors of high‐grade morbidity (*N* = 166).

Variables	AGREE ≥ III	No/Low	OR [95% CI]	*p* (Fish.)	*p* (multi.)
*Procedural factors*					
**LAMS 15 mm**	**12/84 (14.3%)**	27/82 (32.9%)	**0.34 [0.16–0.73]**	**0.006**	**0.004**
Gastric route	31/111 (27.9%)	8/55 (14.5%)	2.28 [0.97–5.36]	0.079	0.051
Double‐pigtail	9/29 (31.0%)	30/137 (21.9%)	1.61 [0.66–3.89]	0.34	—
Cystic duct EUS‐checked	30/129 (23.3%)	9/37 (24.3%)	0.94 [0.40–2.22]	1.00	—
First‐line	7/23 (30.4%)	32/143 (22.4%)	1.52 [0.57–4.01]	0.43	—
LAMS 8 mm	5/23 (21.7%)	34/143 (23.8%)	0.89 [0.31–2.58]	1.00	—
*Patient/disease factors*					
**Ascites**	**8/60 (13.3%)**	31/106 (29.2%)	**0.37 [0.16–0.87]**	**0.023**	**0.040**
Clinical success	27/137 (19.7%)	12/29 (41.4%)	0.35 [0.15–0.81]	0.017	—
ECOG ≥ 3	11/63 (17.5%)	28/103 (27.2%)	0.57 [0.26–1.24]	0.19	—
Male sex	23/82 (28.0%)	16/84 (19.0%)	1.66 [0.80–3.43]	0.20	—
Pancreatic ADK	29/119 (24.4%)	10/47 (21.3%)	1.19 [0.53–2.69]	0.84	—
Duodenal stenosis	19/85 (22.4%)	20/81 (24.7%)	0.88 [0.43–1.80]	0.85	—
Gallstones	5/32 (15.6%)	34/134 (25.4%)	0.54 [0.19–1.53]	0.35	—

*Note:* *N* = 166. Bold: *p* < 0.05.

Abbreviations: Fish., Fisher exact test; Multi., multivariable logistic regression (3 variables: LAMS 15 mm, gastric route, ascites).

## 4 Discussion

This multicenter study represents the largest dedicated analysis of predictors of biliary drainage dysfunction following EUS‐GBD for MDBO. The principal finding is that neither the drainage route (transgastric vs. transduodenal) nor LAMS diameter influenced biliary patency, while ascites was the only independent predictor of dysfunction.

The composite primary endpoint is a methodological strength. Standard patency analysis excludes patients who never achieve clinical success, thereby overestimating efficacy. Our composite definition captures both early failure and delayed obstruction within a single time‐to‐event framework, particularly relevant for predictor identification. Our sensitivity analysis restricted to clinical responders confirmed this, showing no significant predictor of late dysfunction.

The comparable patency between transgastric and transduodenal approaches is a novel finding. Previous literature on EUS‐GBD for cholecystitis suggested a higher risk of food impaction with transgastric LAMS due to gravitational trapping in a dependent position [3, 17, 18]. However, our data demonstrate that 12‐month patency was comparable between routes (68.5% vs. 73.5%, *p* = 0.50) in the jaundice setting. Similarly, LAMS diameter had no influence on patency (*p* = 0.82), contrasting with EUS‐CDS where stent‐to‐duct mismatch has been implicated in dysfunction [12, 19]. The gallbladder's compliance as a drainage reservoir may buffer the effects of stent diameter on bile flow, unlike the relatively rigid common bile duct. Several authors questioned why EUS‐GBD was selected over EUS‐CDS or EUS‐HGS after ERCP failure. In our cohort, the predominant reasons were small common bile duct diameter precluding safe CDS, extensive duodenal invasion preventing stable positioning, insufficient intrahepatic dilation for HGS, or significant ascites. These anatomical constraints, combined with operator expertise, drove the decision. The inclusion of 23 patients (13.9%) who underwent first‐line EUS‐GBD without prior ERCP also deserves comment. This practice reflects emerging prospective evidence supporting EUS‐GBD in palliative MDBO [8, 9], and may have been driven by anticipated ERCP failure, small‐caliber common bile duct, or operator preference. While this heterogeneity is inherent to the retrospective design, first‐line versus rescue indication was not associated with dysfunction (HR 0.76, *p* = 0.61), suggesting the identified predictors apply regardless of indication pathway.

Ascites as the sole independent predictor of dysfunction (HR 2.45, *p* = 0.018) is clinically coherent. Rather than a purely technical impediment, ascites reflects advanced disease biology broadly impacting biliary drainage regardless of technique [20, 21]. Mechanisms include compromised gallbladder wall apposition and progressive malignant infiltration. This underscores that disease biology rather than procedural technique primarily determines long‐term drainage efficacy. Of note, ascites was paradoxically associated with lower high‐grade morbidity (AGREE ≥ IIIa: OR 0.37). This apparent paradox is likely explained by competing risk from early death: patients with ascites had substantially higher mortality (63.3% vs. 47.2%) and shorter median survival (0.8 vs. 3.6 months), reducing their time at risk for procedure‐related morbidity. The reintervention analysis confirmed no significant predictor among responders (ascites HR 2.08, *p* = 0.185), suggesting ascites acts primarily through early clinical failure.

EUS‐confirmed cystic duct patency was independently associated with clinical success (OR 2.88, *p* = 0.019), providing the first multicenter evidence supporting its systematic assessment during EUS‐GBD. A patent cystic duct ensures adequate communication between the gallbladder and the biliary tree, a prerequisite for effective indirect decompression. Our data suggest per‐procedure EUS assessment may add value. However, this should be interpreted cautiously given the retrospective design, non‐standardized assessment, and observer‐level bias. Patients with unverified or obstructed cystic duct should be considered for alternative strategies, pending prospective confirmation.

While LAMS diameter did not influence patency, 15 mm LAMS were independently associated with fewer high‐grade adverse events (OR 0.31, *p* = 0.004). This dissociation between morbidity and patency suggests that larger stent diameter may reduce stent‐related complications (as tissue ingrowth, food impaction, migration) without affecting drainage dynamics. This supports preferential use of 15 mm LAMS when anatomically feasible. However, this finding emerged from an exploratory analysis of cumulative morbidity encompassing both early procedure‐related and late stent‐related events, and should be interpreted with caution. Prospective confirmation in a study designed to evaluate stent size as the primary variable is warranted.

Taken together, our findings suggest that EUS‐GBD is forgiving regarding access route and stent diameter for long‐term drainage. The emphasis should be on: (i) verifying cystic duct patency before completing the procedure; (ii) choosing 15 mm LAMS to minimize morbidity; and (iii) evaluating ascites as a marker of reduced efficacy warranting closer follow‐up.

This analysis identifies factors associated with failure within an EUS‐GBD cohort, not patient selection criteria. Limitations include the retrospective multicenter design and the heterogeneity inherent to the composite endpoint. However, as discussed above, the composite approach was deliberate: it captures both early failure and delayed obstruction, avoids conditioning on an intermediate endpoint, and maximizes statistical power (32 events vs. 22 in the reintervention‐only analysis). Twenty patients with incomplete follow‐up were excluded from survival analyses, potentially introducing selection bias, though their baseline characteristics were comparable. The median follow‐up was 1.7 months overall (2.0 months among censored patients). This short duration reflects the poor prognosis of this palliative cohort, and the 12‐month patency estimate may overestimate true long‐term patency. Center‐level variability could not be fully controlled, but a standardized case report form and the multicenter design mitigate single‐center bias. Cystic duct patency assessment was not protocolized across centers; its predictive value should be confirmed prospectively with a standardized method. Approximately half the cohort overlaps with the CHOLEBLADEUS study [14]; however, research questions, designs, and statistical approaches are entirely distinct, and the present cohort includes additional centers and a longer recruitment period. The limited number of events (*n* = 32) restricted multivariate models to three covariates; prespecified sensitivity analyses and multiple model configurations tested the robustness of findings.

In conclusion, biliary patency after EUS‐GBD for MDBO was not influenced by access route or LAMS diameter. Ascites was the only independent predictor of dysfunction. Cystic duct patency verification was independently associated with clinical success, suggesting its potential value pending prospective confirmation. The use of 15 mm LAMS was associated with fewer high‐grade adverse events without affecting patency, favoring their preferential use when technically feasible.

## Author Contributions

A.D. and M.G.: study concept and design. All participants except T.M.: acquisition of data. A.D., M.G., and T.M.: analysis and interpretation of data. A.D. and M.G.: drafting of the manuscript. All participants: critical revision of the manuscript for important intellectual content. A.D., M.G., and T.M.: approval of the final manuscript. A.D.: guarantor of the article.

## Funding

The authors have nothing to report.

## Conflicts of Interest

A. Debourdeau delivered a lecture sponsored by Boston Scientific. S. Leblanc has received workshop fees from Olympus and Boston. She has received congress fees from Fujifilm. She is on the laboratory board for Alfasigma. B. Napoleon has received fees for teaching from Olympus, Boston Scientific, and MaunaKea (all 2018 to present). N. Williet: honoraria/consulting fees for Accord Healthcare, Astrazeneca, Ipsen, Lac Leo Pharma, Mayoly, MSD, Pierre Fabre, Servier, and Viatris. J. Jacques provides consultancy for Boston Scientific and for Fujifilm (from 2022, ongoing). T. Wallenhorst: Training sessions in endoscopy for Olympus and Fujifilm. J. B. Gornals Consultant for Boston Sc; Fees from Fujifilm.

## Supporting information


**Figure S1:** KM curves restricted to clinical success subgroup, by route (A) and LAMS.
**Figure S2:** KM reintervention‐free survival overall (CS+, *N* = 117, 22 events).
**Figure S3A:** KM reintervention‐free survival by route and LAMS diameter.
**Figure S3B:** KM reintervention‐free survival by ascites status.
**Table S1:** Composite endpoint components by access route.
**Table S2:** Baseline by cystic duct assessment status.
**Table S3:** High‐grade morbidity predictors—early versus late.
**Table S4:** Univariate Cox regression—biliary reintervention (CS+, *N* = 117, 22 events).
**Table S5:** Multivariable Cox regression—biliary reintervention (CS+, *N* = 117).

## Data Availability

The data that support the findings of this study are available on request from the corresponding author. The data are not publicly available due to privacy or ethical restrictions.
