# Supplementary material for: Predictors of Biliary Drainage Dysfunction Following EUS‐Guided Gallbladder Drainage for Malignant Distal Biliary Obstruction: A Multicenter Retrospective Study: GALLBLADEUS‐2
Source: Dig Endosc. 2026 Jul 14;38(7):e70211. doi: 10.1111/den.70211 (PMC13367258; doi:10.1111/den.70211)
Supplement: Supplementary file 1 — Figure S1: KM curves restricted to clinical success subgroup, by route (A) and LAMS. Figure S2: KM reintervention‐free survival overall (CS+, N = 117, 22 events). Figure S3A: KM reintervention‐free survival by route and LAMS diameter. Figure S3B: KM reintervention‐free survival by ascites status. Table S1: Composite endpoint components by access route. Table S2: Baseline by cystic duct assessment status. Table S3: High‐grade morbidity predictors—early versus late. Table S4: Univariate Cox regression—biliary reintervention (CS+, N = 117, 22 events). Table S5: Multivariable Cox regression—biliary reintervention (CS+, N = 117). [file DEN-38-0-s001.docx]

# Supplementary Material

**GALLBLADEUS-2: Supplementary Tables and Figures**

**Supplementary Figure 1.** KM curves restricted to clinical success subgroup, by route (A) and LAMS (B).


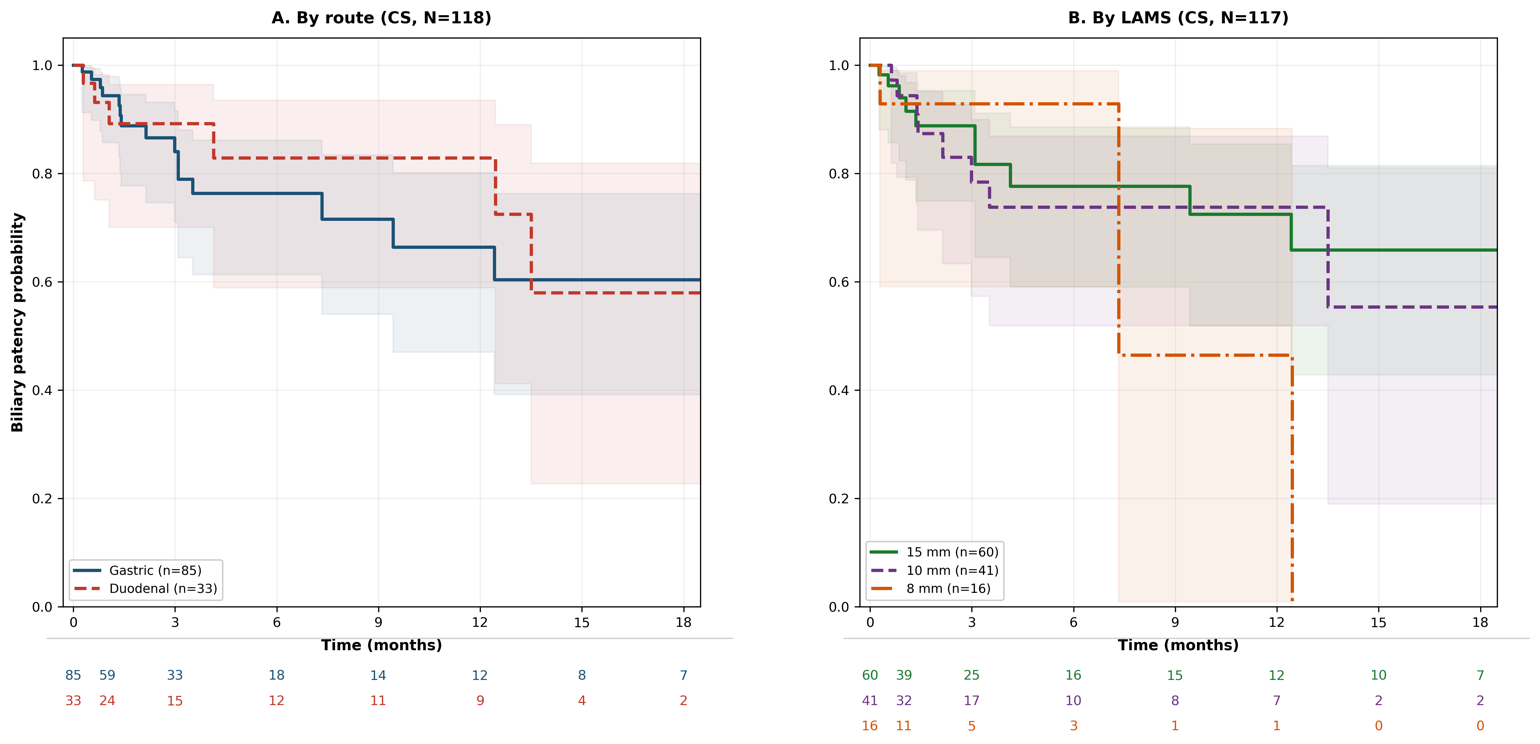


**Supplementary Figure 2.** KM reintervention-free survival overall (CS+, N=117, 22 events).


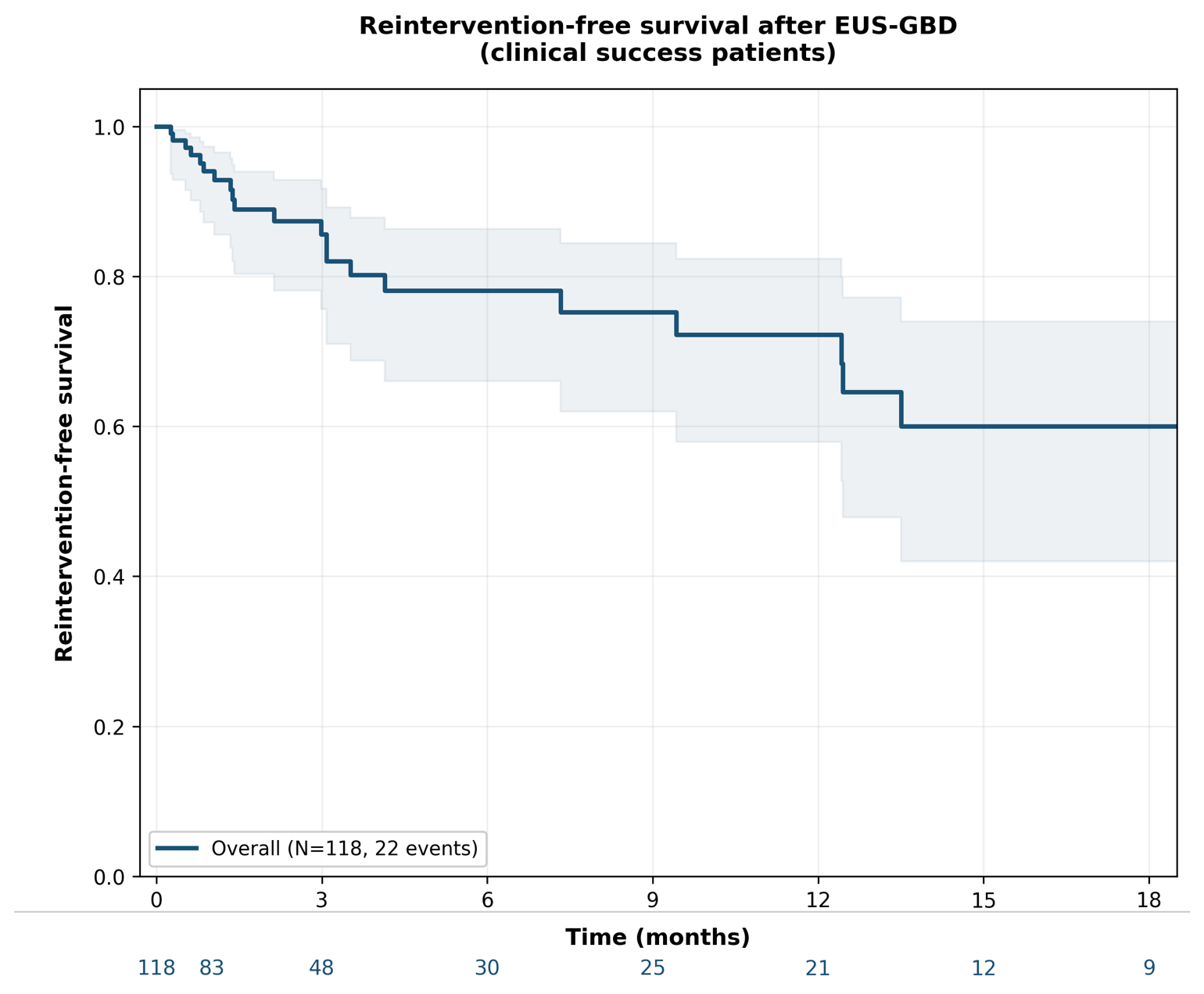


**Supplementary Figure 3A-B.** KM reintervention-free survival by route and LAMS diameter.


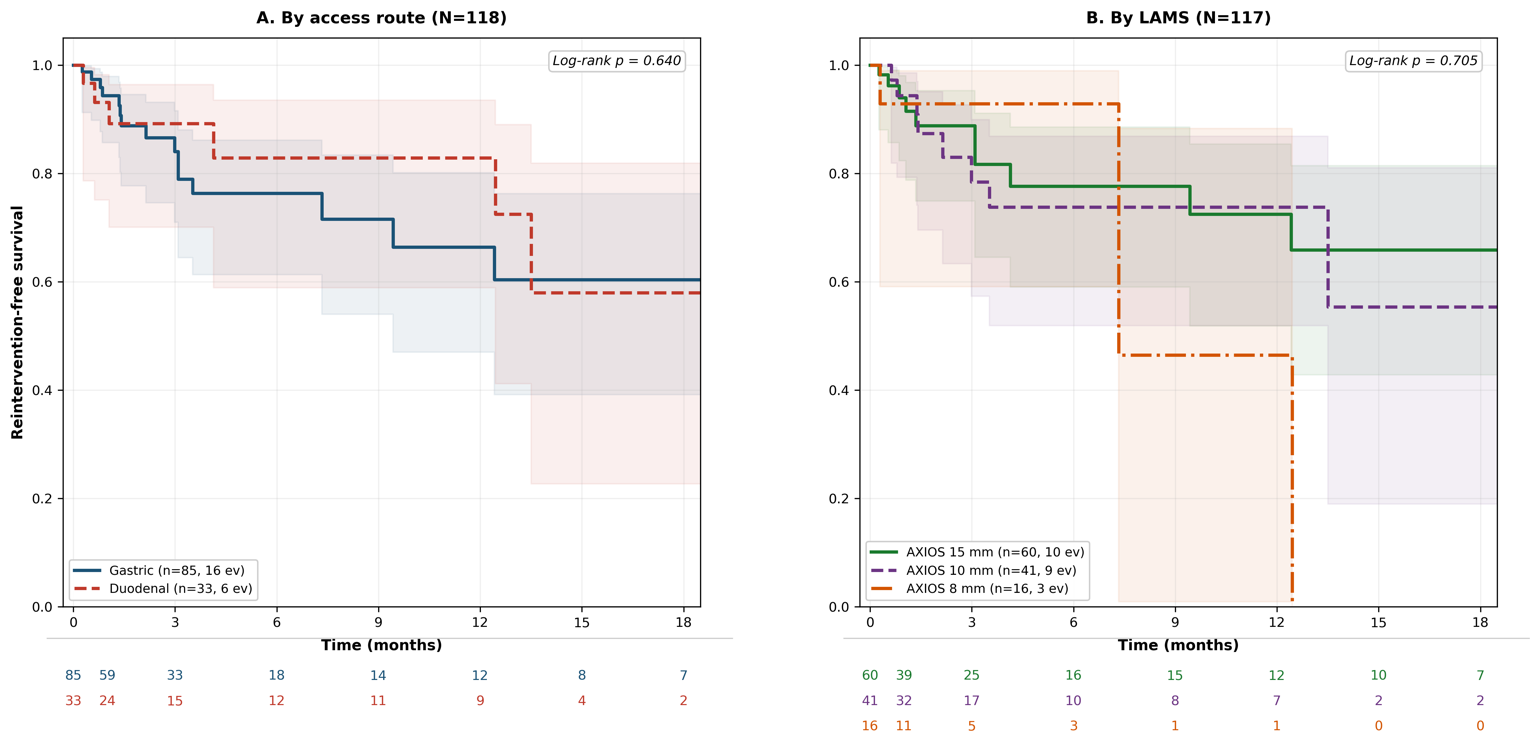


**Supplementary Figure 3B.** KM reintervention-free survival by ascites status.


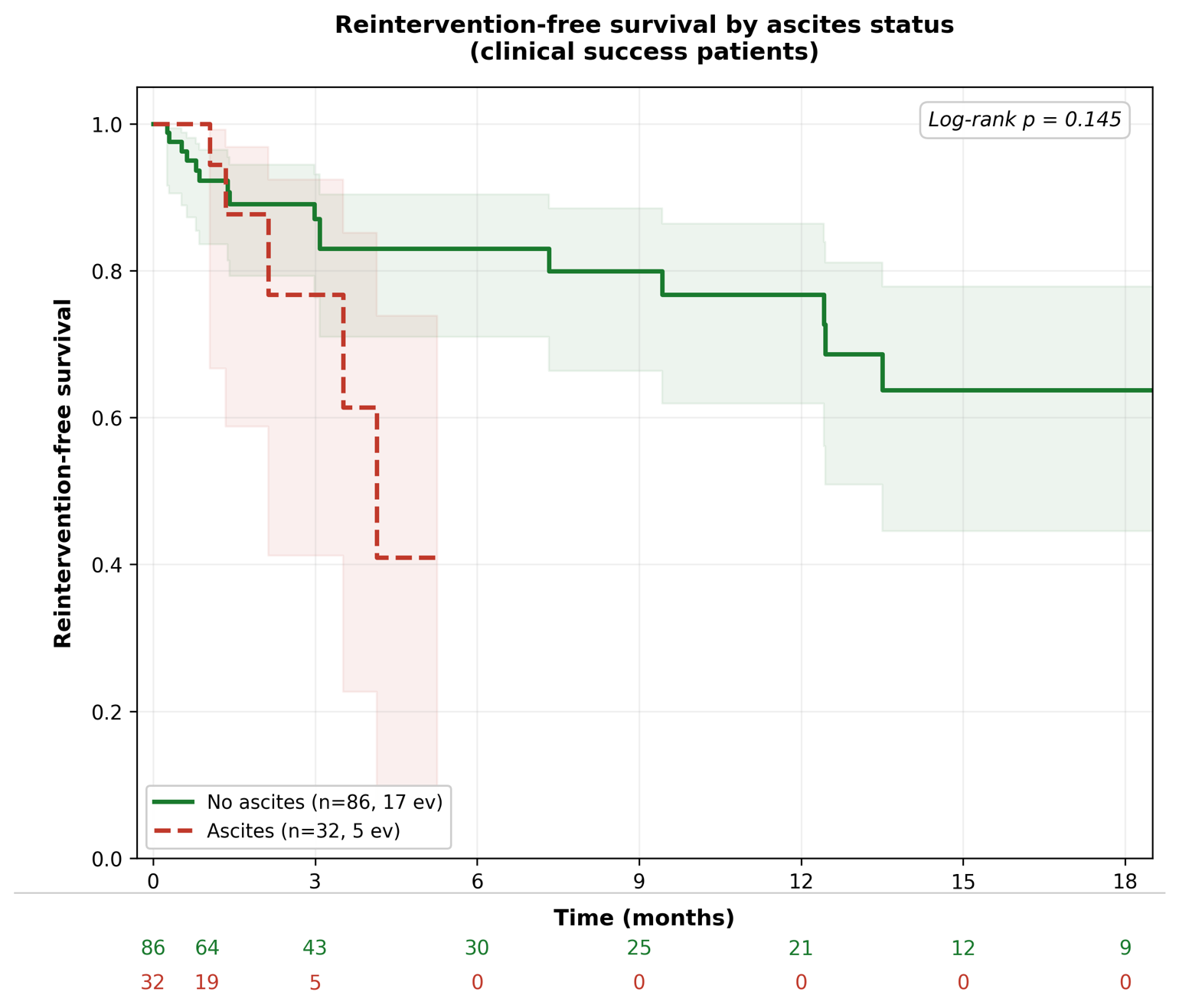


**Supplementary Table 1. Composite endpoint components by access route**

|  | **Transgastric** | **Transduodenal** | **p** |
| --- | --- | --- | --- |
| Early clinical failure | 18/110 (16.4%) | 9/54 (16.7%) | 1.00 |
| Late dysfunction (CS+) | 15/92 (16.3%) | 7/45 (15.6%) | 1.00 |
| Total composite events | 21/103 (20.4%) | 11/43 (25.6%) | 0.54 |

**Supplementary Table 2. Baseline by cystic duct assessment status**

| **Variable** | **EUS-assessed (n=129)** | **Not assessed (n=37)** | **p** |
| --- | --- | --- | --- |
| Age | 71.5 ± 12.4 | 72.8 ± 13.1 | 0.58 |
| Male sex | 64 (49.6%) | 18 (48.6%) | 1.00 |
| Pancreatic ADK | 93 (72.1%) | 26 (70.3%) | 0.84 |
| Metastatic | 69 (53.5%) | 22 (59.5%) | 0.58 |
| Ascites | 45 (34.9%) | 15 (40.5%) | 0.56 |
| Duodenal stenosis | 67 (51.9%) | 18 (48.6%) | 0.85 |
| ECOG ≥3 | 48 (37.2%) | 15 (40.5%) | 0.71 |

**Supplementary Table 3. High-grade morbidity predictors – early vs late**

| **Variable** | **Early ≤30d rate** | **OR [95% CI]** | **Late >30d rate** | **OR [95% CI]** |
| --- | --- | --- | --- | --- |
| LAMS 15 mm | 7/84 (8.3%) | 0.60 [0.22–1.64] | 5/84 (6.0%) | 0.22 [0.08–0.63] |
| Gastric route | 17/111 (15.3%) | 2.10 [0.72–6.14] | 14/111 (12.6%) | 2.65 [0.87–8.12] |
| Ascites | 5/60 (8.3%) | 0.43 [0.15–1.23] | 3/60 (5.0%) | 0.30 [0.08–1.04] |

15 mm LAMS protective effect predominantly driven by late events.

**Supplementary Table 4. Univariate Cox regression – biliary reintervention (CS+, N=117, 22 events)**

| **Variable** | **HR [95% CI]** | **p-value** |
| --- | --- | --- |
| Gastric route | 1.18 [0.46–3.03] | 0.733 |
| LAMS 15 mm | 0.71 [0.31–1.66] | 0.434 |
| Ascites | 2.14 [0.73–6.29] | 0.165 |
| Duodenal stenosis | 1.21 [0.52–2.80] | 0.664 |
| Double-pigtail | 1.42 [0.52–3.89] | 0.493 |
| Cystic duct EUS | 1.70 [0.39–7.30] | 0.477 |
| Gallstones | 0.97 [0.29–3.30] | 0.961 |
| ECOG ≥3 | 0.94 [0.34–2.56] | 0.901 |
| Male sex | 1.56 [0.65–3.72] | 0.316 |
| Pancreatic ADK | 0.80 [0.31–2.07] | 0.651 |
| Metastatic stage | 1.56 [0.67–3.65] | 0.305 |
| First-line | 1.27 [0.43–3.75] | 0.670 |
| Age (per year) | 0.98 [0.95–1.02] | 0.421 |

No variable reached significance.

**Supplementary Table 5. Multivariable Cox regression – biliary reintervention (CS+, N=117)**

| **Variable** | **HR [95% CI]** | **p-value** |
| --- | --- | --- |
| Gastric route | 1.27 [0.49–3.29] | 0.625 |
| LAMS 15 mm | 0.73 [0.31–1.72] | 0.471 |
| Ascites | 2.08 [0.70–6.15] | 0.185 |

Model: route + LAMS + ascites. No variable reached significance.
